# microRNA-204-5p Participates in Atherosclerosis Via Targeting MMP-9

**DOI:** 10.1515/med-2020-0034

**Published:** 2020-03-26

**Authors:** Na Wang, Yuliang Yuan, Shipeng Sun, Guijian Liu

**Affiliations:** 1 Guang’anmen Hospital, Chinese Academy of Chinese Medical Science, No. 5 North Line Pavilion, Xicheng District, Beijing 100053, China; 2 Clinical laboratory, Guang’anmen Hospital Southern District, Chinese Academy of Chinese Medical Science, Beijing 102618, China

**Keywords:** Atherosclerosis, miR-204-5p, MMP-9, VSMCs, proliferation, apoptosis, migration

## Abstract

The aim of the present study was to investigate the role and mechanism of microRNA-204-5p (miR-204-5p) in atherosclerosis (AS)-related abnormal human vascular smooth muscle cells (hVSMCs) function. Firstly, we analyzed the expression of miR-204-5p and found that the miR-204-5p expression level was clearly downregulated in atherosclerotic plaque tissues and blood samples compared to the normal controls. Then, matrix metallopeptidase-9 (MMP-9) was predicted to be the potential target of miR-204-5p by TargetScan and this prediction was confirmed by luciferase assays. Besides, we observed that miR-204-5p could negatively regulate the expression of MMP-9 in hVSMCs. Subsequently, Thiazolyl Blue Tetrazolium Bromide (MTT) assay, transwell assay and flow cytometry were performed to detect the proliferation, migration and apoptosis of hVSMCs. Down-expression of miR-204-5p led to the promotion of proliferation and migration accompanied with the suppression of apoptosis in hVSMCs, and these effects were reversed by MMP-9-siRNA. In addition, overexpressed miR-204-5p could inhibit hVSMC proliferation and migration and promote the apoptosis of hVSMCs. However, the effects were also abrogated by overexpressed MMP-9. Together, our findings showed that miR-204-5p plays an important role in the growth and migration of hVSMCs by targeting MMP-9, which might be a novel biomarker and promising therapeutic target for AS.

## 1 Introduction

Atherosclerosis (AS), a chronic and devastating lesion characterized by a cumulation of fibrous elements and lipids in large arteries, leads to most of the high morbidity and mortality globally [1]. The occurrence of atherosclerotic lesions is usually originated with endothelial dysfunctions followed by invasion of macrophages, proliferation and migration of smooth muscle cells [2]. Recently, it was reported that vascular smooth muscle cells (VSMCs) played an essential part in AS development [3] and the abnormal proliferation and migration of VSMCs facilitated the formation, amplification, and restructuring of AS [4]. Thus, it is urgent and significant to explore a predominant therapeutic strategy to suppress proliferation and migration of VSMCs for the treatment of AS.

MicroRNAs (miRNAs) are a group of small, short and noncoding RNA molecules, which are extensively involved in adjusting gene expressions through interfering with miRNA-mRNA interaction [5]. Recent researches have demonstrated that miRNAs regulate a variety of fundamental biological progress such as inflammation [6], tumor, as well as take part in the evolution of AS [7]. Numerous studies have showed that the expression levels of miRNA are considered to be involved in the proliferation, migration, and phenotypic of VSMCs [8]. A prior study indicated that miR-145 regulated the VSMCs phenotypic switching in AS [9]. In addition, Xu et al. found that miR-499a-3p promoted the proliferation and migration of VSMCs through straightly targeting myocyte enhancer factor 2C (MEF2C) in AS [10]. However, despite the above researches, the effects of many other miRNAs shown in VSMCs still need to be explored.

Matrix metalloproteinases (MMPs), a group of more than 26 neutral zinc-dependent endopeptidases [11], are known to be associated with normal vessels remodeling [12], wound healing [13] and inflammation [14]. Recently, MMPs were found in atherosclerotic tissues, and MMP-9 was particularly evident in unstable atherosclerotic plaques while weak in stable atherosclerotic plaques [15]. Previous studies also indicate that the expression level of MMP-9 was high in the weak regions of atherosclerotic

plaques and this might be due to the rupture of plaque [16]. Furthermore, it was reported that miR-204 was able to adjust the proliferation and invasion of retinoblastoma cells through a direct targeting of the MMP-9 gene [17]. Considering a regulated role of miRNAs in AS and the relationship between miRNAs and MMP-9 in proliferation and invasion, we hypothesized that miRNAs might act as an important regulator in the progress of AS by targeting MMP-9.

In this study, we aimed to investigate the role and mechanism of miR-204-5p in the growth and migration of hVSMCs, so as to explore the role of miR-204-5p in AS *in vitro*. This study might offer a novel role of miR-204-5p in the pathogenesis of AS and thus provide innovative therapeutic strategy for atherosclerotic patients.

## 2 Materials and methods

### 2.1 Clinical specimens collection

A total of 30 patients with AS (age range, 41-59 years; male/ female, 15/15) and 30 healthy subjects (age range, 43-58 years; male/female: 15/15) were enrolled in this study. The atherosclerotic plaque tissues and corresponding normal plaque tissues were collected from atherosclerotic patients. Peripheral blood was received from 30 patients and 30 healthy subjects, and then centrifuged at 1,2000 g at 4°C for 8 min to separate serum. All specimens were separated and immediately snap-frozen in liquid nitrogen until analysis. A protocol for this study was approved by the Ethics Committee of the Guang’anmen Hospital, Chinese Academy of Chinese Medical Science and informed consents were obtained from all participants.

### 2.2 Cell culture and transfection

The Human VSMCs were obtained from American Tissue Culture Collection (ATCC, USA). The hVSMCs were cultured in Dulbecco’s Modified Eagle Medium (DMEM) supplemented with 10% fetal bovine serum (FBS, SiJi-Qing, Hangzhou, China) and 1% penicillin-streptomycin. Then, the cells were kept in a humidified incubator of 5% CO_2_ at 37°C. The miR-204-5p inhibitor, miR-204-5p mimic, inhibitor control, and mimic control were obtained from GenePharma (Shanghai, China). MMP-9-plasmid, control-plasmid, MMP9-siRNA and control-siRNA were obtained form Santa Cruz. Then, the miRNAs and plasmids were transfected into VSMCs using Lipofectamine^TM^ 2000 transfection reagent (Invitrogen) in line with the manufacturer’s protocol.

### 2.3 MiRNA target analysis and dual-luciferase reporter assay

TargetScan 7.2 (http://www.targetscan.org/vert_72/) was applied to predict the potential target of miR-204-5p and MMP-9 was identified as a potential target gene of miR-204-5p. According to the bioinformatics results, the wild-type (WT) and mutant (MUT) seed regions of miR-204-5p in the 3’-untranslated region (UTR) of MMP-9 gene were synthesized *in vitro* and then inserted into a pmirGLO vector (Promega Corporation, Madison, WI, USA). Then, hVSMC cells were seeded in triplicate in 24-well plates and transfected with luciferase reporter plasmids and mimic control or miR-204-5p mimic using Lipofectamine 2000 reagent (Invitrogen). After transfection for 48 h, the luciferase signals were examined using the Dual Luciferase Reporter Assay System (Promega) and the results were normalized to the Renilla luciferase activity.

### 2.4 qRT-PCR

Total RNA was extracted from hVSMCs, tissue lysates or blood samples using Trizol reagent (Invitrogen, Thermo Fisher Scientific, Inc.). The purity and concentrations of RNA were determined by Nanodrop ND1000 instrument. cDNA was obtained using HiScript^TM^ II Q RT SuperMix for qPCR (Vazyme Biozyme Co., Ltd) from 1 μg RNA and the temperature protocol was as follows: 42˚C for 2 min, 50˚C for 15 min and 85˚C for 5 sec. Amplification was performed using qRT-PCR in a Step one plus system (Roche Molecular Diagnostics, Pleasanton, CA, USA) using ChamQ^TM^ Universal SYBR qPCR Master Mix (Vazyme). The reaction protocol was as follows: 30 cycles of denaturing at 95˚C for 60 sec, annealing at 60°C for 60 sec, and chain extension at 72˚C for 1 min, followed by a final extension step at 72˚C for 10 min. The primer sequences were as follows:

GAPDH forward, 5′-CTTTGGTATCGTGGAAGGACTC-3′;

reverse, 5′-GTAGAGGCAGGGATGATGTTCT-3′;

U6 forward, 5′-GCTTCGGCAGCACATATACTAAAAT-3′;

reverse, 5′-CGCTTCACGAATTTGCGTGTCAT-3′;

miR-204-5p forward, 5′-ACACTCCAGCTGGGTTCCCTTTGT-CATCCTAT-3′;

reverse, 5′-CTCAACTGGTGTCGTGGA-3′;

MMP-9 forward, 5′-AGACCTGGGCAGATTCCAAAC-3′;

MMP-9 reverse, 5′-CGGCAAGTCTTCCGAGTAGT-3′.

GAPDH or U6 were applied as an internal control and relative quantification method (2^−***δδC***q^) [18] was carried out to quantify relative expression levels.

### 2.5 Western blot analysis

The hVSMCs, tissue lysates and blood samples were treated with radioimmunoprecipitation lysis buffer (Invitrogen, CA, USA), and centrifuged at 12,000 rpm and 4*˚C* for 15 min to obtain the total proteins. The supernatant was applied to detect protein concentration using the Protein Assay Kit (Beyotime, Haimen, China). Protein samples were separated by 10% Sodium dodecylsulphate polyacrylamide gel electrophoresis (SDS-PAGE) at 100 V and then transferred onto polyvinylidene fluoride (PVDF) membranes. After transfer, the membranes were blocked with 5 g/L skimmed milk at room temperature for 1 h and subsequently incubated with MMP-9 (cat. no. 13667; dilution rate: 1:1000; Cell Signaling Technology, Inc.) and GAPDH (cat. no. 5174; dilution rate: 1:1000; Cell Signaling Technology, Inc.) overnight. Following washing with phosphate buffered solution-tween-20 (PBST) four times of 10 min, the membranes were incubated with corresponding horseradish peroxidase (HRP)-linked secondary antibody (cat. no. 7074; 1:2,000; Cell Signaling Technology, Inc.) for 1 h at room temperature. Finally, membranes were washed four times with PBST for 10 min and developed using the chemiluminescence (ECL) plus Kit (Pierce, USA).

### 2.6 MTT assay

The proliferation of hVSMCs was determined by the MTT assay according to the manufacturer’s protocol. Briefly, the cells were seeded into 96-well plates at a density of 10,000 cells per well 24 h prior to transfection. After transfection for 48 h, MTT solution (25 μL) was added to each well and cultivated at 37°C for 4 h. After discarding the supernatant, 200 μL dimethyl sulfoxide (DMSO) was added and slightly shaken for 10 min to dissolve the formazan crystals. Lastly, the 96-well plates were placed at a micro-plate reader (Molecular Devices, Sunny- vale, CA, USA) and the extinction was measured at 490 nm.

### 2.7 Flow cytometry analysis

After transfected for 48 h, hVSMCs were trypsinized and then centrifuged at 1000 rpm for 5 min at room temperature. Furthermore, the sedimentary cells were re-suspended and adjusted at a density of 1×10^6^ cells/ml. Subsequently the cells were stained with Annexin-V-fluorescein isothiocyanate (FITC) and propidium iodide (PI) for 15 min in dark. After staining, the cell apoptosis was analyzed by flow cytometry (Calibur; BD, USA), and data on cell apoptosis was collected analyzed using FlowJo software (version 10.0).

### 2.8 Transwell assay

The hVSMCs were starved through culturing in serum-free medium for 12 h. Then, cells were transfected with inhibitor control, miR-204-5p inhibitor, mimic control, miR-204-5p mimic, miR-204-5p inhibitor+control-siRNA, miR-204-5p inhibitor+MMP-9-siRNA, miR-204-5p mimic+control-plasmid or miR-204-5p mimic+MMP-9-plasmid. After 48 hours of transfection, cells were harvested and the migratory ability was detected using a Transwell chamber. In brief, 1×10^5^ hVSMCs were seeded into the upper chamber pre-coated with or without Matrigel (BD Biosciences). The lower chambers were filled with 400 *μL* medium containing 10% FBS. After incubation with 5% CO_2_ at 37°C for 16 hours, non-migrated or no-invaded cells were removed from the on the topside of the filter by cotton bud. Then, the migrated or invaded cells were washed with PBS and fixed with 4% paraformaldehyde solution and stained with 0.1% crystal violet for half an hour at room temperature, imaged, and counted. Each sample was performed in triplicate.

### 2.9 Statistical analysis

Statistical analysis was performed with SPSS v16.0 (SPSS, Chicago, IL, USA). Measurement data were expressed as the mean ± standard deviation (SD) of 3 independent experiments. Student’s t-test was used to comparing the data between two groups. The one-way analysis of variance with Tukey’s post hoc test was used for comparisons of multiple samples. p<0.05 was considered to indicate a statistically significant difference.

## 3 Results

### 3.1 miR-204-5p was downregulated in atherosclerotic tissues and blood

**Figure 1:**
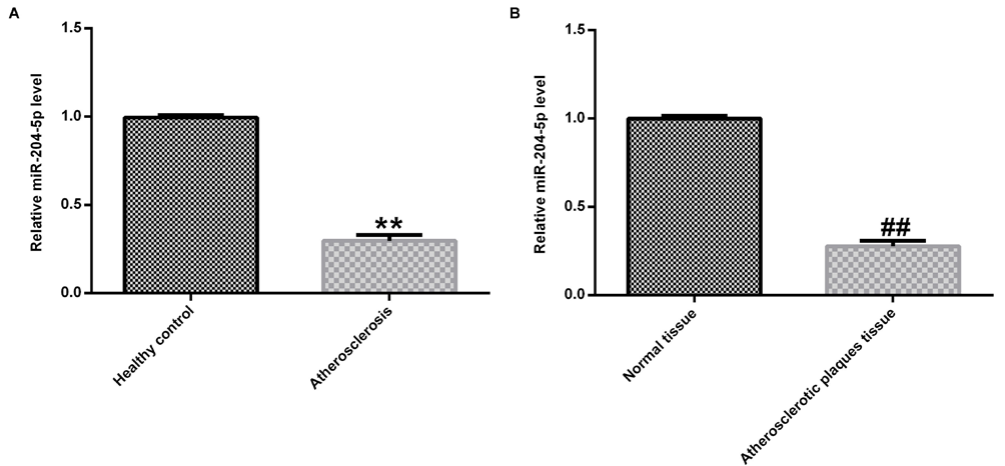
The low expression of miR-204-5p in atherosclerotic tissues and blood. (A) The expression levels of miR-204-5p in blood from 30 atherosclerotic patients and 30 healthy subjects were determined by qRT-PCR assay. (B) The expression levels of miR-204-5p in 30 atherosclerotic plaque tissues and 30 normal control tissues were detected by qRT-PCR assay. Data were exhibited as mean ± SD.**p < 0.01.

In order to detect the activity of miR-204-5p in the progression of AS, qRT-PCR assay was conducted to investigate the miR-204-5p expression level in atherosclerotic tissues and blood. Generally, the miR-204-5p expression in atherosclerotic blood samples was decreased in comparison to those the in healthy individuals (Figure 1A). In the same way, the atherosclerotic plaque tissues had lower miR-204-5p expression than adjacent tissues (Figure 1B).

### 3.2 MMP-9 was a direct target of miR-204-5p

**Figure 2:**
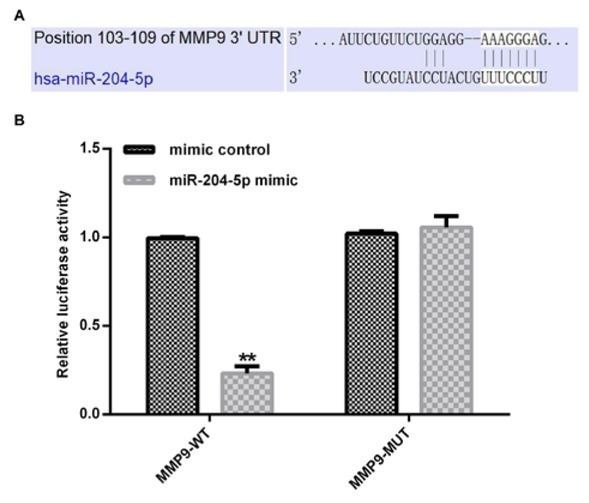
miR-204-5p directly targets MMP-9. (A) The position of miR-204-5p target sites in 3′-UTR of MMP-9. (B) Dual-luciferase assay was used to confirm the binding sites between miR-204-5p and MMP9. Each value represents as means ± SD. **p < 0.01.

In order to understand the molecular mechanisms of miR-204-5p in AS, prediction program TargetScan was used to foresee the potential targets of miR-204-5p. As shown in Figure 2A, we found that MMP-9 was a candidate target of miR-204-5p. To further verify the predicted binding sites between MMP-9 and miR-204-5p, luciferase reporter constructs were performed. In Figure 2B, miR-204-5p markedly reduced the luciferase activity of MMP-9 wild-type 3’-UTR compared with the mimic control group, while no obvious effect on MMP-9 3’-UTR-mut reporter. Taken together, we concluded that MMP-9 was a direct target of miR-204-5p.

### 3.3 miR-204-5p negatively regulated the expression of MMP-9 in hVSMCs

**Figure 3:**
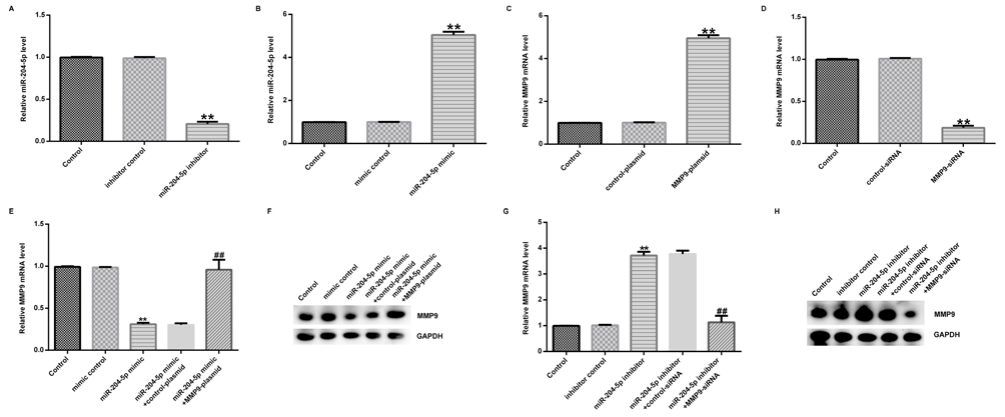
miR-204-5p regulated the expression of MMP-9 in hVSMCs. (A) After transfected with miR-204-5p inhibitor and inhibitor control, the expression of miR-204-5p in hVSMCs was detected by qRT-PCR assay. (B) After transfected with miR-204-5p mimic and mimic control, the expression level of miR-204-5p in hVSMCs was determined by qRT-PCR assay. (C) After transfected with MMP-9-plasmid and control-plasmid, the mRNA expression level of MMP-9 in hVSMCs was determined by qRT-PCR assay. (D) After transfected with MMP9-siRNA and control-siRNA, the mRNA expression level of MMP-9 in hVSMCs was determined by qRT-PCR assay. (E and F) The expressions of MMP-9 were detected by qRT-PCR and Western blot assay in miR-204-5p mimic accompanied by control-plasmid or MMP-9-plasmid groups at both mRNA and protein levels. (G and H) The expressions of MMP-9 were detected by qRT-PCR and Western blot assay in miR-204-5p inhibitor accompanied by control- siRNA or MMP-9-siRNA groups at both mRNA and protein levels. Data presented were the mean ± SD. **p < 0.01 vs. Control group; ## p < 0.01 vs. miR-204-5p mimic/inhibitor group.

To determine the effect of miR-204-5p on the expression of MMP-9 in hVSMCs, hVSMCs were transfected with inhibitor control, miR-204-5p inhibitor, miR-204-5p mimic, mimic control, MMP-9-plasmid, control-plasmid, MMP9-siRNA and control-siRNA. After transfected for 48 h, qRT-PCR analysis was performed to detect the transfection efficiency. The results showed that the expression level of miR-204-5p was lower in miR-204-5p inhibitor transfected group compared to the control group in hVSMCs (Figure 3A). In addition, the miR-204-5p expression was higher in miR-204-5p mimic group than that in the control group (Figure 3B). Furthermore, the expression of MMP-9 was significantly upregulated in MMP-9-plasmid transfected hVSMCs compared with the control group (Figure 3C). Meanwhile, the MMP-9 expression was downregulated after transfected with MMP9-siRNA compared with the control group (Figure 3D). Additionally, the expression of MMP-9 was reduced by miR-204-5p mimic in hVSMCs compared to the control at both mRNA and protein levels, while the effect was reversed by MMP-9-plasmid (Figure 3E and F). Consistently, the expression of MMP-9 was promoted by miR-204-5p inhibitor and compared to the control and the effect was also reversed by MMP-9-siRNA (Figure 3G and H). Collectively, the above results demonstrated that miR-204-5p could negatively adjust the expression of MMP-9 in hVSMCs.

### 3.4 Downregulation of MMP-9 abrogated the promotion of miR-204-5p inhibitor on hVSMC growth and migration

**Figure 4:**
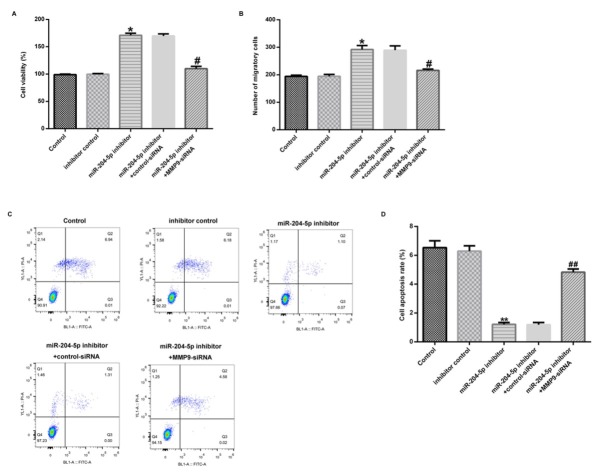
Downexpression of MMP-9 abrogated the promotion of miR-204-5p inhibitor on hVSMC growth and migration. hVSMCs were respectively transfected with inhibitor control, miR-204-5p inhibitor alone or accompanied with control-siRNA or MMP-9-siRNA for 48 h. (A) MTT assay was used to detect cell viability of hVSMCs. (B) The transwell assay was performed to check cell migration. (C and D) hVSMC apoptosis was detected by flow cytometric assay. Results were presented as mean ± SD. *, **p < 0.05, 0.01 vs. Control group; #, ## p < 0.05, 0.01 vs. miR-204-5p inhibitor group.

MMP-9 was considered as to be regulated by miR-204-5p in hVSMCs, we further investigated whether MMP-9 was involved in miR-204-5p - mediated regulations in hVSMCs. Firstly, hVSMCs were transfected with inhibitor control, miR-204-5p inhibitor alone or accompanied with control-siRNA or MMP-9-siRNA for 48 h. After transfection, MTT assay, flow cytometry assay and transwell analysis were conducted to detect cell viability, apoptosis and migration respectively. As shown in Figure 4A, downregulation of miR-204-5p resulted in increased cell viability in hVSMCs. In Figure 4B, transwell analysis demonstrated that downregulation of miR-204-5p promoted the migratory activity of hVSMCs. Results from flow cytometry assay showed that downregulation of miR-204-5p inhibited the cell apoptosis of hVSMCs (Figure 4C and D). However, all these functions were reversed by MMP-9- siRNA.

### 3.5 Overexpression of MMP-9 abrogated the inhibition of miR-204-5p upregulation on hVSMC growth and migration

**Figure 5:**
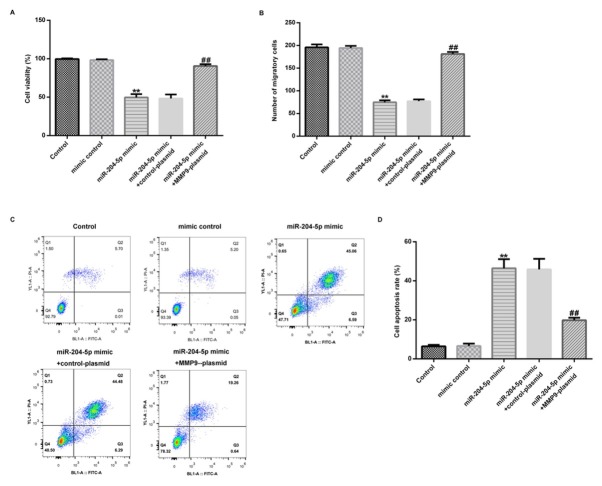
Overexpression of MMP-9 abrogated the inhibition of miR-204-5p mimic on hVSMC growth and migration. hVSMCs were respectively transfected with mimic control, miR-204-5p mimic alone or accompanied with control-plasmid or MMP-9-plasmid for 48 h. (A) MTT assay was used to detect cell viability of hVSMCs. (B) The transwell assay was performed to check cell migration. (C and D) hVSMC apoptosis was detected by flow cytometric assay. Results were presented as mean ± SD. **p < 0.01 vs. Control group; ## p < 0.01 vs. miR-204-5p mimic group.

Based on the previous experiment, we further explored the crucial role of overexpressed MMP-9 on miR-204-5p-mediated regulations of cell growth and migration in hVSMCs. In brief, mimic control, miR-204-5p mimic alone or accompanied with control-plasmid or MMP-9-plasmid were transfected into hVSMCs for 48 h. The results demonstrated that miR-204-5p upregulation evidently inhibit the viability (Figure 5 A) and migration (Figure 5 B) in hVSMCs, while promoted apoptosis in hVSMCs (Figure 5 C and D). However, all the above effects were inverted by MMP-9-plasmid. Together, the results indicated that miR-204-5p targeted MMP-9 and regulated the proliferation, apoptosis and migration of hVSMCs.

## 4 Discussion

Atherosclerosis (AS), a chronic and multifactorial lesion, is the pathological base of many kinds of cardiac and cerebral vascular illness and kept with chronic inflammatory responses [19]. It has been reported that the aberrant growth and migration of VSMCs are associated with the development of AS [20]. Thus, it is necessary to identify the molecular mechanisms underlying the dedication of VSMCs to AS, which will help to find treatment strategies for controlling AS. Recently, accumulating researches have demonstrated that miRNAs provide novel guidelines to studying the development and progression of AS. It was showed that miR-204-5p were associated with the pathogenesis of different kinds of tumor, such as hepatocellular carcinoma [21], colorectal cancer [22] and prostate cancer [23]. Previous study also identified that suppression of miR-204-5p could increase human choriocarcinoma (JAR) cell growth, reduce cell apoptosis with cell cycle changes and thus was considered to be a potential biomarker for the prevention and therapy of pregnancy-induced hypertension (PIH) [24]. However, the biological role of miR-204-5p and its molecular mechanisms in AS still remains uncertain.

In the present study, the expression of miR-204-5p was identified in atherosclerotic tissues and blood using qRT-PCR and the results showed that miR-204-5p was lower expressed in atherosclerotic plaque tissues and blood compared to the control. Moreover, we confirmed that MMP-9 was a direct target of miR-204-5p by TargetScan and Dual-luciferase assay illustrated that miR-204-5p downregulated the MMP-9 wild-type 3’-UTR activity. In the past few years, MMP-9 has emerged as new potential biomarkers of AS. Prior study demonstrated that the expression levels of MMP-9 in serum from patients with carotid AS instability were obviously upregulated [25]. In this study, we found that the expression of MMP-9 was also upregulated in atherosclerotic plaque tissues and blood.

The finding was in agreement with the above research indicating an involvement of MMP-9 in the progress of AS.

In order to identify whether MMP-9 was regulated by miR-204-5p during the process of VMSCs, we transfected inhibitor control, miR-204-5p inhibitor, miR-204-5p mimic, mimic control, MMP-9-plasmid, control-plasmid, MMP9-siRNA and control-siRNA into VMSCs. Results from western blot and q-PCR assay revealed that miR-204-5p mimic obviously decreased the expression of MMP-9 at both protein level and mRNA level, while the suppression was reversed by MMP-9-plasmid. Similarly, miR-204-5p inhibitor was able to increase the MMP-9 expression at both protein and mRNA levels and the promotion was also inversed by MMP-9-siRNA.

Previous studies have identified the important role of miRNAs in the regulation of VSMCs growth and migration processes. Additionally, MMP-9 was considered to be able to promote the VSMCs migration. Thus, we further investigated the relationship between miR-204-5p and MMP-9 on the growth and migration in VSMCs. Results from the present study showed that downregulation of miR-204-5p might contribute to the promotion of proliferation and migration and the inhibition of apoptosis in VSMCs. However, the downexpression of MMP-9 reversed the miR-204-5p inhibitor-mediated functions in VSMCs. In addition, overexpressed miR-204-5p could inhibit VSMCs proliferation and migration and promote the apoptosis of VSMCs. However, the effects were also abrogated by overexpressed MMP-9. These results demonstrated that miR-204-5p could regulate the growth and migration of VSMCs through targeting MMP-9, which revealed a probable pathway for its affection in AS.

Taken together, our results indicated that miR-204-5p regulated the proliferation, apoptosis and migration of hVSMCs, which were related to AS relying on targeting MMP-9. Therefore, our findings might offer a new therapeutic and diagnostic strategy for the prevention and treatment of AS. However, there is a big difference between *in vitro* studies and the real human AS. Moreover, this study represents only *in vitro* preliminary study on the role of miR-204-5p in AS. In order to make our conclusions more convincing, *in vivo* experiments should be performed. These are limitations of this study and further research is needed.
